# Utilization trends for endoscopic ablation therapy and esophagectomy in Barrett’s esophagus from 2005 to 2019

**DOI:** 10.1038/s41598-022-21838-5

**Published:** 2022-10-21

**Authors:** Arvind J. Trindade, Jianying Zhang, Kara L. Raphael, Jiejing Qiu, John Hauschild, Petros C. Benias

**Affiliations:** 1 Division of Gastroenterology, Long Island Jewish Medical Center, Zucker School of Medicine at Hofstra/Northwell, Northwell Health System, 270-05 76th Avenue, New Hyde Park, NY 11040 USA; 2 Institute of Health Innovations and Outcomes Research, Feinstein Institutes for Medical Research, Northwell Health, Manhasset, NY USA; 3 Medtronic Health Economics and Outcomes Research Department, Medical Surgical Portfolio, Minneapolis, USA; 4 Medtronic Health Economics, Policy & Reimbursement Department, Gastrointestinal Operating Unit, Minneapolis, USA

**Keywords:** Gastroenterology, Oesophagogastroscopy

## Abstract

Guidelines have shifted to now recommend endoscopic eradication therapy for Barrett’s esophagus (BE) with low and high-grade dysplasia. Previously, esophagectomy was the standard therapy for high-grade dysplasia. However, it is unclear to what degree ablation therapy has affected utilization of esophagectomy. In this retrospective observational cohort study of BE patients without cancer from the Premier Healthcare Database, the prevalence of utilization of endoscopic ablation therapy and of esophagectomy in BE were calculated and temporal trends were evaluated. A total of 938, 333 BE cases were included in the study. There was a significantly increasing trend of ablation over the period 2006 to 2010 (Annual Percentage Change (APC); 95% CI 0.56% [0.51%, 0.61%]), a significantly decreasing trend for the period 2011 to 2015 (APC; 95% CI − 0.15% [− 0.20%, − 0.11%]), and a shallow increasing trend for the period 2016 to 2019 (APC; 95% CI 0.09% [0.06%, 0.11%]). For esophagectomy, there was a significantly decreasing trend for the period 2006 to 2009 (APC; 95% CI − 0.03% [− 0.04%, − 0.02%]; *P* < 0.001) that corresponded to the uptrend in utilization of endoscopic ablation. There was a stable trend of esophagectomy over the period 2010 to 2019 (APC; 95% CI − 0.0006% [− 0.0002%, 0.0005%]; *P* = 0.1947). Adoption and increased utilization of endoscopic ablation therapy for BE has coincided with a decrease in esophagectomy, and is the predominate method of therapy for BE with dysplasia.

## Introduction

Barrett’s esophagus (BE) is a precancerous condition in which normal squamous esophageal mucosa is replaced by specialized intestinal metaplasia^1^. It is a well-known risk factor for esophageal adenocarcinoma (EAC). BE is prevalent in 1–2% of the population. BE is thought to develop into cancer in a step-wise fashion from low-grade dysplasia to high-grade dysplasia, and eventually to EAC^2^. Endoscopic eradication therapy (ERT) is indicated for patients with dysplasia and superficial cancer (T1a)^1,3^. ERT consists of endoscopic resection and ablation. Endoscopic resection is indicated for raised superficial lesions followed by ablation of the remaining Barrett’s segment. Currently available ablation platforms are radiofrequency ablation (RFA) and cryotherapy^3–5^. Currently, esophagectomy is reserved for patients with cancer staged T1b (sm2 and 3) and higher, dysplastic BE not amenable to endoscopic therapy (i.e. bulky raised disease not amenable to endoscopic resection), or persistent dysplastic BE: ractory to endoscopic therapy. However, prior to the development of ERT, patients with high-grade dysplasia were also treated with esophagectomy^6^.

Although esophagectomy is a definitive way of curing BE and associated dysplasia, it can bring considerable morbidity and mortality. The mortality rate is 2% and the morbidity rate is 10%^7–9^. Operative complications can include anastomotic leaks, respiratory complications, and swallowing problems. Thus, avoiding esophagectomy is an advantage of ERT. It should be noted that outcomes of esophagectomy have never been compared to outcomes to ERT in a prospective randomized fashion. Given the comorbidities associated with esophagectomy, such a trial is not expected^7^.

The goal of endoscopic ablation is for complete eradication of intestinal metaplasia to prevent the formation of cancer^1^. Prospective studies have shown that ablation therapy after removal of raised lesions by endoscopic resection can prevent the progression to cancer in dysplastic BE^3–5^. This should correlate to a decrease in esophagectomy rates. However, there is limited real-world data examining esophagectomy rates for therapy in BE and it’s comparison during pre-ERT to post ERT eras. It is unclear if the esophagectomy rate for BE is truly declining. The aim of this study is to compare pre-ERT to post-ERT esophagectomy utilization in comparison to ablation therapy utilization in a large healthcare database.

## Methods

### Study design and data source selection

We conducted a retrospective observational cohort study using the Premier Healthcare Database (PHD, Charlotte, North Carolina, USA). Informed consent from each patient in the database is not possible due to lack of no identifying information available. The study protocol conforms to the ethical guidelines of the 1975 Declaration of Helsinki.

The PHD is a large, U.S. hospital-based, service-level, all-payer database. Inpatient admission data include over 127 million visits with more than 11 million per year since 2012, representing approximately 25 percent of annual U.S. inpatient admissions. Outpatient encounters include over 947 million outpatient visits with more than 102 million visits per year since 2012. Outpatient visits to emergency departments, ambulatory surgery centers and alternate sites of care are included. The PHD contains data from over 244 million unique patients. Patients can be tracked in the same hospital across the inpatient and hospital-based outpatient settings. There are more than 700 hospitals providing data yearly since 2012^10^. In terms of data quality, for most data elements, less than 1% of patient records have missing information, and for key elements, such as demographics and diagnostic information, less than 0.01% have missing data^11^.

This database was chosen for its scope, as it includes both inpatients and outpatients. This allows for estimation of the prevalence of (1) all BE patients in a single year within the database; (2) endoscopic ablation procedures (usually outpatient procedures); and (3) surgical esophagectomies (which require an inpatient recovery). The underlying diagnosis of BE in the database population was integral to determine the utilization rate of endoscopic ablation and esophagectomy in BE.

Based on data availability, 14 years (2005–2019) of PHD data were used. The PHD is considered exempt from Institutional Review Board oversight as dictated by Title 45 Code of Federal Regulations, Part 46 of the United States, specifically 45 CFR 46.101(b)(4). In accordance with the HIPAA Privacy Rule, disclosed data from Premier are considered de-identified per 45 CFR 164.506(d)(2)(ii)(B) through the “Expert Determination” method. The study was exempt from the Zucker School of Medicine at Hofstra/Northwell institutional board review due to the database containing no patient identifiable information. The need for informed consent was waived by the Zucker School of Medicine at Hofstra/Northwell institutional board review due to the retrospective nature of the study and lack of patient identifying information in the study. The study protocol conforms to the ethical guidelines of the 1975 Declaration of Helsinki and followed the Strengthening the Reporting of Observational Studies in Epidemiology (STROBE) reporting guideline.

### Cohort selection

To calculate the prevalence of utilization of ERT and esophagectomy among patients with BE, we included all adult patients who were diagnosed with BE each year of the study period. A patient with BE was identified by using International Statistical Classification of Diseases the 9th revision (ICD-9-CM) or the 10th revision (ICD-10-CM) diagnosis codes of BE (Appendix 1). ERT was identified by using the Current Procedural Terminology (CPT®) codes (Appendix 1). Esophagectomy was identified by using International Statistical Classification of Diseases the 9th revision (ICD-9-CM) or the 10th revision (ICD-10-PCS) surgical codes. We only included patients with a primary diagnosis of BE for these procedures. Billing codes for radiofrequency ablation and cryotherapy were not used as they are the same and thus cannot be differentiated within a dataset.

A patient with BE was counted once during a one-year period. The same patient could be counted again in subsequent years if this patient had at least one visit with a BE diagnosis during that year and met selection criteria. To this end, we could capture all BE patients who could potentially receive ERT or esophagectomy during the study period. When a patient had multiple hospital visits during a one-year period, the first visit that had a BE diagnosis was considered the index diagnosis. CY2006 was the first year in which an index BE diagnosis was made. We used the first year’s available data (CY2005) as a buffer to apply to our cohort selection criteria. Patients were excluded if they (1) had a diagnosis of esophageal or stomach cancer 6 months prior to and during the year of index diagnosis; (2) had an esophageal ablation procedure within 12 months prior to index diagnosis; (3) had an endoscopic resection on the same day as an esophageal ablation procedure. The rationale for these exclusion criteria are as follows: (1) early versus advanced esophageal or gastric cancer could not be differentiated in this database, and as curative ablation therapy is inappropriate for advanced cancer, only patients with BE and low or high grade dysplasia without cancer were included; (2) ERT for BE should be performed only after the index diagnosis is made; (3) endoscopic resection and ablation for BE do not usually occur on the same day.

### Outcome measures

The primary outcome was to determine the utilization of esophageal ablation and esophagectomy among patients with BE without cancer in the United States. The prevalence of ERT and of esophagectomy were evaluated on a yearly basis during the study period. The secondary outcome was to identify differences in patient demographics and hospital characteristics among BE patients who underwent ERT versus those who underwent esophagectomy.

### Statistical analysis

Univariate analysis was used to compare patient demographics and hospital characteristics. Chi-square test was used for categorical variables. The prevalence and associated 95% CIs in utilization of ablation and esophagectomy in each year was calculated. To evaluate temporal trends in utilization of ablation, both the unadjusted relative risk (RR) and adjusted relative risk (aRR) for annual change were calculated, as linear trends in utilization of ablation and esophagectomy across the years of our study period were not observed. These analyses were performed using generalized estimating equation (GEE) models with a robust covariance estimator to adjust for provider (hospital) clustering effect. The index BE diagnosis year was treated as a categorical variable and the year with the highest prevalence of utilization of the procedure was selected as a reference year (for ERT - CY2010, for esophagectomy - CY2006).

To calculate the aRR of ablation, a priori–specified covariates were used, including age, sex, race, marital status, health insurance type, hospital bed size, regions, teaching hospital and urban hospital. To assess temporal trends in utilization of esophagectomy, the adjusted relative risk (aRR) was calculated by adjusting for provider (hospital) clustering effect only. Due to the extremely low number of esophagectomy cases in the study cohort, a best-fit model with the priori–specified covariates was not able to be found. Statistical significance was two-tailed and was set at *P* < 0.05. Data preparation and analyses were performed using SAS software (version 9.4; SAS Institute, Inc., Cary NC, USA.

## Results

**Figure 1:**
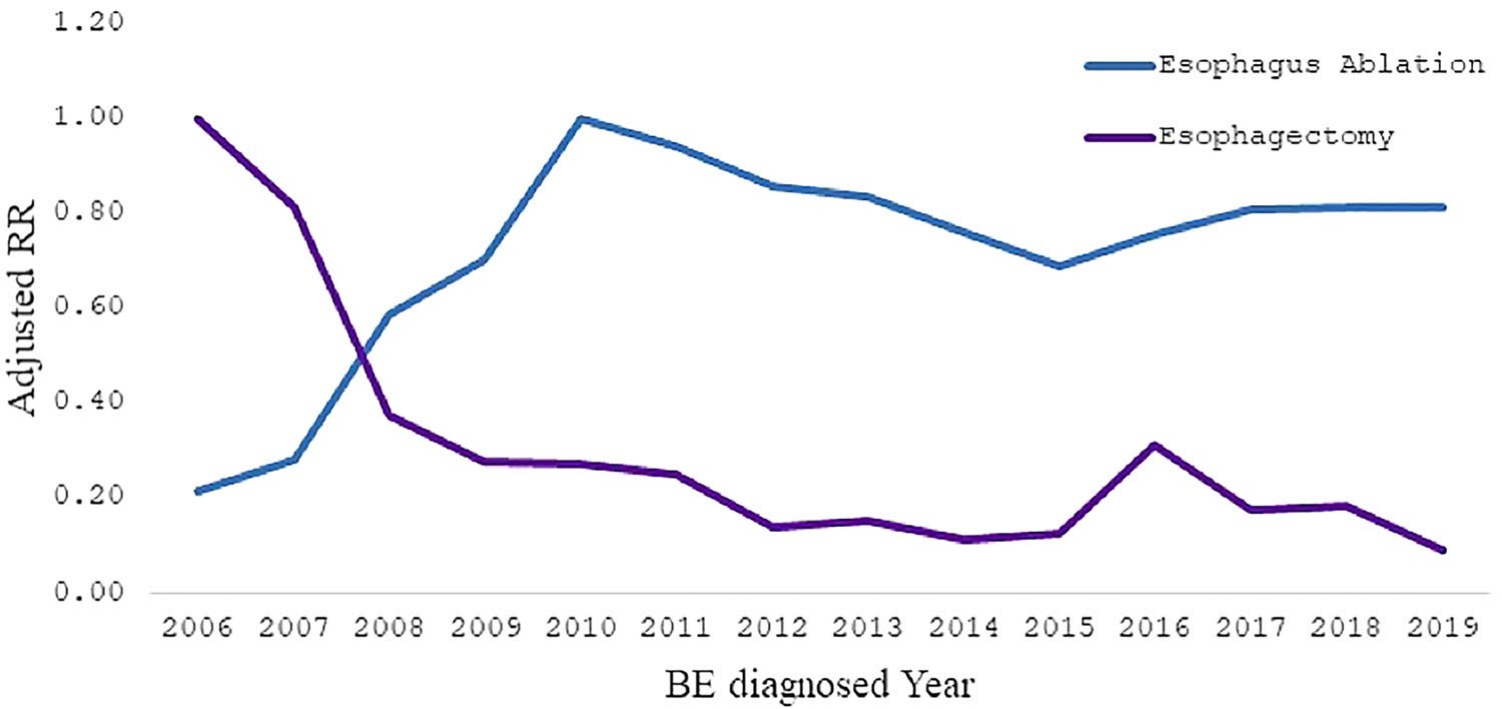
Trends in ablation versus esophagectomy from 2006 to 2019.

A total of 938, 333 patients with BE were included in the study. The overall prevalence of ERT and esophagectomy for BE was 2018 per 100,000 and 20 per 100,000 BE patients, respectively (Table 1). The use of ERT increased from 626 (95% CI 530–735) per 100,000 BE patients in 2006 to 2019 (95% CI 1983–2158) per 100,000 BE patients in 2019. Overall, there was a significantly increasing trend for ERT over the period 2006 to 2010 (Annual Percentage Change (APC); 95% CI 0.56% [0.51%, 0.61%]), a significantly decreasing trend for the period 2011 to 2015 (APC; 95% CI − 0. 15% [− 0.20%, − 0.11%]), and a shallow increasing trend for the period 2016 to 2019 (APC; 95% CI 0.09% [0.06%, 0.11%]) (Fig. 1).

**Table 1:** Prevalence of esophagus ablation and Esophagectomy in Barrett’s esophagus patients.

Year	Barrett’s Esophagus	Esophagus Ablation		Esophagectomy	
	N	n	%	n	%
2006	23,795	150	0.63	25	0.10
2007	26,627	215	0.80	22	0.08
2008	31,673	522	1.62	12	0.04
2009	36,761	708	1.89	10	0.03
2010	46,491	1288	2.70	12	0.03
2011	60,421	1553	2.51	13	0.02
2012	69,761	1600	2.24	8	0.01
2013	79,688	1802	2.21	10	0.01
2014	86,546	1761	1.99	8	0.01
2015	94,765	1715	1.78	10	0.01
2016	91,789	1834	1.96	25	0.03
2017	94,190	2024	2.10	15	0.02
2018	95,716	2041	2.09	15	0.02
2019	100,110	2115	2.07	7	0.01
All	938,333	19,328	2.02	192	0.02

The values represent the annual number of cases esophagus ablation and Esophagectomy per 10,000 Barrett’s esophagus cases.

During the same time period of increased utilization of ERT, the utilization of esophagectomy decreased from 104 (95% CI 68–154) per 100,000 BE patients in 2006 to 7 (95% CI 3–14) per 100,000 BE patients in 2019.

Overall, there was a significantly decreasing trend for esophagectomy for the period 2006 to 2009 (APC; 95% CI − 0.03% [− 0.04%, − 0.02%]; *P* < 0.001), which corresponded to the uptrend in utilization of ERT. There was a stable trend for esophagectomy over the period 2010 to 2019 (APC; 95% CI − 0.0006% [− 0.0002%, 0.0005%]; *P* = 0.1947) (Table 2 and Fig. 1).

**Table 2:** Comparison of patient and hospital characteristics of patients who underwent an intervention versus those that did not.

	Esophagus ablation				*p*-value	Esophagectomy				*p*-value
	No		Yes			No		Yes		
	n	%	n	%		n	%	n	%	
**Age group (Year)**					< 0.0001					0.0036
18–34	22,638	2.41	302	1.56		22,939	2.4	1	0.52	
35–44	54,980	5.86	917	4.74		55,882	5.84	15	7.81	
45–54	141,640	15.09	2879	14.9		144,476	15.09	43	22.4	
55–64	238,335	25.40	5402	27.95		243,681	25.45	56	29.17	
≥ 65	480,740	51.23	9828	50.85	< 0.0001	490,491	51.23	77	40.1	
**Gender**										< 0.0001
Female	399,029	42.53	5423	28.06		404,402	42.24	50	26.04	
Male	539,116	57.45	13,903	71.93		552,877	57.74	142	73.96	
Unknown	188	0.02	2	0.01	< 0.0001	190	0.02			
**Race/Ethnicity**										0.6938
Caucasian	786,611	83.83	16,507	85.4		802,958	83.86	160	83.33	
Black	26,043	2.78	353	1.83		26,393	2.76	3	1.56	
Hispanic	32,558	3.47	794	4.11	< 0.0001	33,345	3.48	7	3.65	
Others	93,121	9.92	1674	8.66		94,773	9.9	22	11.46	
**Marital status**										0.6813
Married	509,364	54.28	11,948	61.82		521,208	54.44	104	54.17	
Single	343,115	36.57	5479	28.35	< 0.0001	348,527	36.4	67	34.9	
Others	85,854	9.15	1901	9.84		87,734	9.16	21	10.94	
**Insurance type**										0.0141
Medicare	509,343	54.28	9697	50.17		518,957	54.2	83	43.23	
Medicaid	65,061	6.93	860	4.45		65,902	6.88	19	9.9	
Commercial	312,089	33.26	7817	40.44	< 0.0001	319,825	33.4	81	42.19	
Others	51,840	5.52	954	4.94		52,785	5.51	9	4.69	
**Hospital bed size**										< 0.0001
000–099	79,364	8.46	676	3.5		80,039	8.36	1	0.52	
100–199	161,710	17.23	1861	9.63		163,565	17.08	6	3.13	
200–299	168,081	17.91	2562	13.26		170,626	17.82	17	8.85	
300–399	142,856	15.22	2500	12.93		145,336	15.18	20	10.42	
400–499	144,364	15.39	3532	18.27	< 0.0001	147,861	15.44	35	18.23	
500+	241,958	25.79	8197	42.41		250,042	26.11	113	58.85	
**Hospital region**										0.009
Midwest	269,427	28.71	5412	28		274,797	28.7	42	21.88	< 0.0001
Northeast	148,291	15.80	2082	10.77		150,334	15.7	39	20.31	
South	361,483	38.52	9501	49.16	< 0.0001	370,895	38.74	89	46.35	
West	159,132	16.96	2333	12.07	< 0.0001	161,443	16.86	22	11.46	
Teaching hospital	404,716	43.13	9341	48.33		413,926	43.23	131	68.23	< 0.0001
Urban hospital	799,502	85.20	17,781	92		817,104	85.34	179	93.23	0.0018

Chi-square test was used to compare the difference between patients with and without esophagus ablation or esophagectomy.

Patient and hospital characteristics of each group are summarized in Table 3. Patients with BE who underwent ERT were more likely to be between 55 and 64 years old (25.4% vs. 27.9%, *P* < 0.0001), male (57.5% vs. 71.9%, *P* < 0.0001), Hispanic (3.5% vs. 4.1%, *P* < 0.0001), married (54.3% vs. 61.8%, *P* < 0.0001), and have commercial health insurance (33.3% vs. 40.4%, *P* < 0.0001) than patients with BE that did not undergo ERT. Furthermore, patients treated in hospitals with 500 or more beds (25.8% vs. 42.4, *P* < 0.0001), who lived in the Southern region (38.5% vs. 49.2%, *P* < 0.0001) and were treated at a teaching hospital (43.1% vs. 48.3%, *P* < 0.0001) were also more likely to undergo esophagus ablation than no ERT.

**Table 3:** Patient and hospital characteristics.

	Ablation		Esophagectomy		*P* value
	n	%	n	%	
**Age group (Year)**					0.0022
18–34	302	1.56	1	0.52	
35–44	915	4.74	15	7.81	
45–54	2876	14.89	43	22.4	
55–64	5399	27.95	56	29.17	
≥ 65	9828	50.87	77	40.1	
**Gender**					0.8381
Female	5422	28.06	50	26.04	
Male	13,896	71.93	142	73.96	
Unknown	2	0.01			
**Race/Ethnicity**					0.5618
Caucasian	16,500	85.4	160	83.33	
Black	353	1.83	3	1.56	
Hispanic	793	4.1	7	3.65	
Others	1674	8.66	22	11.46	
**Marital status**					0.0931
Married	11,943	61.82	104	54.17	
Single	5478	28.35	67	34.9	
Others	1899	9.83	21	10.94	
**Insurance type**					0.0023
Medicare	9695	50.18	83	43.23	
Medicaid	859	4.45	19	9.9	
Commercial	7812	40.43	81	42.19	
Others	954	4.94	9	4.69	
**Hospital bed size**					< 0.0001
000–099	676	3.5	1	0.52	
100–199	1861	9.63	6	3.13	
200–299	2561	13.26	17	8.85	
300–399	2500	12.94	20	10.42	
400–499	3530	18.27	35	18.23	
500+	8192	42.4	113	58.85	
**Region**					0.0006
Midwest	5409	28	42	21.88	
Northeast	2081	10.77	39	20.31	
South	9498	49.16	89	46.35	
West	2332	12.07	22	11.46	
Teaching hospital	9333	48.31	131	68.23	< 0.0001
Urban hospital	17,774	92	179	93.23	0.5561

Chi-square test was used to compare the difference between patients who had esophagus ablation and who had esophagectomy.8 patients who had ablation then had esophagectomy were grouped into esophagectomy group.

Patients with BE who underwent esophagectomy were more likely to be between 55 and 64 years old (*P* = 0.0036), male (57.7% vs. 74.0%, *P* < 0.0001), and have commercial health insurance (33.4% vs. 44.2%, *P* = 0.0095) than patients with BE that did not undergo esophagectomy. Patients who were treated in hospitals with 500 or more beds (26.1% vs. 58.9, *P* < 0.0001), who lived in the Southern region (38.7% vs. 46.4%, *P* = 0.0077) and were treated at a teaching hospital (43.2% vs. 68.2%, *P* < 0.0001) were also more likely to have an esophagectomy than no esophagectomy (Table 3).

Finally, patients with BE who were between 35 and 54 years old (*P* = 0.0022), had Medicaid health insurance (4.4% vs. 9.9%, *P* = 0.0022), were treated in hospitals with 500 or more beds (42.4% vs. 58.9%, *P* < 0.0001), who lived in the Northeast region (10.8% vs. 20.3%, *P* = 0.0003) and were treated at a teaching hospital (48.3% vs. 68.2%, *P* < 0.0001) were more likely to have esophagectomy than to undergo ERT.

## Discussion

In this study we examined the utilization trends in endoscopic ablation and esophagectomy for management of BE without cancer. We found that increased utilization of ERT correlated with a decrease in the utilization of surgical esophagectomy. Based on these trends, it can be inferred that endoscopic ablation therapy has helped decrease utilization of surgical esophagectomy for management of BE without cancer. To our knowledge this is the first study that has shown these trends. Although it was previously assumed that ablation therapy decreased esophagectomy rates, it was unclear if this truly was the case as real world practices in the management of BE were not previously assessed.

We found that the increased use of ERT occurred primarily between 2007 and 2010. While the pivotal trial that demonstrated the success of RFA in the treatment of BE was formally published in 2009^3^, the interim results were initially presented in 2008. In addition, there was a multicenter US registry on the use of RFA that was published in 2008^12^. Use of RFA for the treatment of BE began prior to these publications and the trials began in 2007, which corresponds to the observed increased uptick of ERT in this study period.

This study also aimed to examine if there were any patient or hospital factors that predicted the use of ERT versus esophagectomy for management of BE without cancer. We found similar patient characteristics for both patients undergoing ERT and esophagectomy, likely reflecting the typical patient population that requires therapy for BE (male, middle aged, from the southern region, treated in teaching hospitals, and those with commercial insurance). However, we did find some risk factors that did predispose patients to esophagectomy over endoscopic ablative therapy. These included younger patients (age 35–54), patients with Medicaid insurance, those treated in larger hospitals (> 500 beds), and treatment in the Northeast region. Younger patients are generally better surgical candidates, and thus this may be the reason for this risk factor. It is unclear why the other risk factors predispose to esophagectomy. Without individual patient details, it is difficult to examine why these may be risk factors.

It should be noted that although ERT decreases the need for upfront surgery, esophagectomy is still part of the armamentarium in the management of BE. Esophagectomy still should be utilized for patients with refractory strictures either intrinsic to BE or iatrogenic due to endoscopic therapy; for dysplastic BE refractory to an array of ablation modalities, or for bulky raised BE not amenable to resection. Our data shows that surgery is still being utilized, although at a low rate (Fig. 1, Table 1). Furthermore, although ERT is safe, there are still small risks associated with it including bleeding, perforation, and stricture formation. ERT also requires multiple sessions to eradicate the BE and requires ongoing endoscopic surveillance after complete eradication, and carries the risk of interval cancers. Although this study shows the positive effects of endoscopic ablation therapy of BE in the United States, the rate of esophageal cancer continues to rise in the United States^13,14^. In fact, of about 10,000 esophageal adenocarcinomas diagnosed each year in the USA, only about 7% are identified through current screening approaches^13^. Most esophageal adenocarcinoma is being diagnosed at a late stage in which ERT or esophagectomy cannot be offered to the patient. Thus, improved screening tools and non-invasive devices are needed to better screen for BE with dysplasia^15–21^. Endoscopic ablation therapy will not be fully utilized to its potential without enhancing our approach to better screen for Barrett’s with dysplasia.

Our study has several strengths. It uses a large database of BE patients that capture both inpatient and outpatient billing procedure codes and diagnoses; in addition, patients can be tracked between procedures. Thus we are able to estimate utilization of both ERT and esophagectomy for BE. We use the same database to determine the number of patients diagnosed with BE each year to estimate esophagectomy and ERT rates rather than report the crude number of procedures performed for each modality. This allows for a more accurate estimation of endoscopic or surgical therapy utilization for management of BE. In addition, we exclude cancer cases to truly gauge how BE with low-grade and high-grade dysplasia is being managed. Including cancer cases in the dataset would create a heterogeneous group, as the dataset cannot differentiate between superficial (T1a) and invasive (T2 and above) esophageal cancer. In addition, the dataset cannot differentiate between squamous cell cancer and adenocarcinoma (the cancer for which BE is the major risk factor); and is thus another reason to exclude BE patients with cancer.

Our study does have limitations. This study estimates the utilization trends of ERT and esophagectomy using one large database. It does not capture every procedure in the USA and thus does not report on the incidence of these procedures. In addition, like any study of this nature, it relies on accurate billing and diagnosis codes. Finally, the database cannot distinguish between BE with dysplasia and nondysplastic BE. It is assumed that most BE without cancer undergoing therapy is dysplastic BE per the national guidelines^1,22^. In addition, the database cannot distinguish between radiofrequency ablation and other forms of ablation therapy (e.g., cryotherapy and photodynamic therapy) as they are all billed under the same code. Although RFA is the most utilized ablation therapy and is currently the only recommended therapy in the guidelines for eradication of dysplastic BE, photodynamic therapy was the first ablation modality^1,22,23^.

In conclusion, BE ablative therapy has been shown to be an effective tool to eradicate dysplasia^3,5^. This study shows that with increasing ablation therapy utilization, the rates of esophagectomy for BE decreased. As discussed, further efforts need to be aimed at better screening for BE with dysplasia to utilize the full potential of ablation therapy in BE.

## Supplementary Information

Supplementary Information.

## Data Availability

The dataset used for this study will be provided upon request as allowed per Premier Database user guidelines. Requests can be directed to the corresponding author.

## Abbreviations

BE: Barrett’s esophagus
APC: Annual percentage change
EAC: Esophageal adenocarcinoma
RFA: Radiofrequency ablation
PHD: Premier Health Database
