# Supplementary material for: Utilization trends for endoscopic ablation therapy and esophagectomy in Barrett’s esophagus from 2005 to 2019
Source: Sci Rep. 2022 Oct 21;12:17619. doi: 10.1038/s41598-022-21838-5 (PMC9587253; doi:10.1038/s41598-022-21838-5)
Supplement: Supplementary file 1 — Supplementary Information. [file 41598_2022_21838_MOESM1_ESM.docx]

Appendix 1: Codes to identify the study population and procedures

|  | **ICD_9 diagnosis codes** | **ICD_10 diagnosis codes** |  |
| --- | --- | --- | --- |
| Barrett’s esophagus | 530.85 Barrett’s esophagus 230.1 Carcinoma in-situ of esophagus | K22.70 Barrett's esophagus without dysplasia K22.710 Barrett's esophagus with low grade dysplasia K22.711 Barrett's esophagus with high grade dysplasia K22.719 Barrett's esophagus with dysplasia, unspecified  D00.1 carcinoma in-situ of esophagus |  |
| Esophageal Cancer | 150.0 malignant neoplasm of cervical esophagus 150.1 malignant neoplasm of thoracic esophagus 150.2 malignant neoplasm of abdominal esophagus 150.3 malignant neoplasm of upper third of esophagus 150.4 malignant neoplasm of middle third of esophagus 150.5 malignant neoplasm of lower third of esophagus  150.8 malignant neoplasm of other unspecified part of esophagus 150.9 malignant neoplasm of esophagus, unspecified site | C15.3 Malignant neoplasm of upper third of esophagus  C15.4 Malignant neoplasm of middle third of esophagus  C15.5 Malignant neoplasm of lower third of esophagus  C15.8 Malignant neoplasm of overlapping sites of esophagus  C15.9 Malignant neoplasm of esophagus, unspecified |  |
| Stomach Cancer | 151.0 Malignant neoplasm of cardia  151.1 Malignant neoplasm of pylorus 151.2 Malignant neoplasm of pyloric antrum 151.3 Malignant neoplasm of fundus of stomach 151.4 Malignant neoplasm of body of stomach 151.5 Malignant neoplasm of lesser curvature of stomach, unspecified  151.6 Malignant neoplasm of greater curvature of stomach 151.8 Malignant neoplasm of other specified sites of stomach  151.9 Malignant neoplasm of stomach, unspecified site | C16.0 Malignant neoplasm of cardia  C16.1 Malignant neoplasm of fundus of stomach  C16.2 Malignant neoplasm of body of stomach  C16.3 Malignant neoplasm of pyloric antrum  C16.4 Malignant neoplasm of pylorus  C16.5 Malignant neoplasm of lesser curvature of stomach, unspecified  C16.6 Malignant neoplasm of greater curvature of stomach, unspecified  C16.8 Malignant neoplasm of overlapping sites of stomach  C16.9 Malignant neoplasm of stomach, unspecified |  |
|  | **ICD_9 Surgical Procedure codes** | **ICD_10 Surgical Procedure codes** | **CPT codes** |
| Esophagectomy | 42.10 Esophagostomy, not otherwise specified  42.11 Cervical esophagostomy  42.40 Esophagectomy, not otherwise specified  42.41 Partial esophagectomy  42.42 Total esophagectomy | 0DT10ZZ Resection of Upper Esophagus, Open Approach 0DT14ZZ Resection of Upper Esophagus, Percutaneous Endoscopic Approach 0DT17ZZ Resection of Upper Esophagus, Via Natural or Artificial Opening  0DT18ZZ Resection of Upper Esophagus, Via Natural or Artificial Opening Endoscopic 0DT20ZZ Resection of Middle Esophagus, Open Approach 0DT24ZZ Resection of Middle Esophagus, Percutaneous Endoscopic Approach 0DT27ZZ Resection of Middle Esophagus, Via Natural or Artificial Opening 0DT28ZZ Resection of Middle Esophagus, Via Natural or Artificial Opening Endoscopic 0DT30ZZ Resection of Lower Esophagus, Open Approach  0DT34ZZ Resection of Lower Esophagus, Percutaneous Endoscopic Approach 0DT37ZZ Resection of Lower Esophagus, Via Natural or Artificial Opening 0DT38ZZ Resection of Lower Esophagus, Via Natural or Artificial Opening Endoscopic 0DT50ZZ Resection of Esophagus, Open Approach 0DT54ZZ Resection of Esophagus, Percutaneous Endoscopic Approach 0DT57ZZ Resection of Esophagus, Via Natural or Artificial Opening 0DT58ZZ Resection of Esophagus, Via Natural or Artificial Opening Endoscopic |  |
| Esophagus Ablation |  |  | 43228 Esophagoscopy, with ablation of tumor(s), polyp(s), or other lesion(s), not amenable to removal by hot biopsy forceps, bipolar cautery or snare technique  43229 Esophagoscopy, flexible, transoral; with ablation of tumor(s), polyp(s), or other lesion(s) (includes pre- and post-dilation and guide wire passage, when performed)  43258 Esophagoscopy, rigid or flexible; diagnostic with ablation of tumor(s), polyp(s), or other lesion(s) not amenable to removal by hot biopsy forceps, bipolar cautery or snare technique  43270 Esophagogastroduodenoscopy, flexible, transoral; with ablation of tumor(s), polyp(s), or other lesion(s) (includes pre- and post-dilation and guide wire passage, when performed) |
| Endoscopic resection of nodular |  |  | 43211 Esophagoscopy, flexible, transoral; with endoscopic mucosal resection 43216 Esophagoscopy, flexible, transoral; with removal of tumor(s), polyp(s), or other lesion(s) by hot biopsy forceps  43217 Esophagoscopy, flexible, transoral; with removal of tumor(s), polyp(s), or other lesion(s) by snare technique  43250 Esophagogastroduodenoscopy, flexible, transoral; with removal of tumor(s), polyp(s), or other lesion(s) by hot biopsy forceps  43251 Esophagogastroduodenoscopy, flexible, transoral; with removal of tumor(s), polyp(s), or other lesion(s) by snare technique  43254 Esophagogastroduodenoscopy, flexible, transoral; with EMR (endoscopic mucosal resection) |
